# 

**Published:** 1888-01

**Authors:** 


### THE SOUTHERN MEDICAL RECORD.

EDITORS:

T. S. POWELL, M. D. R. C. WORD, M. D.

COLLABORATORS:

J. McF. GASTON, M. D.,
W. D. BIZZELL, M. D.,
W. P. NICOLSON, M. D.,
E. VAN GOIDTSNOVEN, M. D.,
V. O. HARDON, M. D.,
G. G. ROY, M. D.,
A. G. HOBBS, M. D.,
W. S. ELKIN, M. D.,
W. A. CROW, M. D.,
JNO. THAD. JOHNSON, M. D.

**Terms: $2.00 per Annum, in Advance; 4 Copies $6.00; Single Copies 20 Cents.**

Address R. C. WORD, M.D., Managing Editor, Atlanta, Ga.

Published and Entered as Second-class Matter at ATLANTA, GA.

